# Household Air Pollution from Cooking Fuels Increases the Risk of Under-Fives Acute Respiratory Infection: Evidence from Population-Based Cross-Sectional Surveys in Tanzania

**DOI:** 10.5334/aogh.3650

**Published:** 2022-06-29

**Authors:** Festo K. Shayo, Deogratius Bintabara

**Affiliations:** 1 Tanrep Research Consultancy Limited, P.O Box 31147, Dar es Salaam, TZ; 2 Muhimbili University of Health and Allied Sciences, P.O Box 65001, Dar es salaam, TZ; 3 Department of Community Medicine, School of Medicine and Dentistry, The University of Dodoma, Dodoma, TZ

**Keywords:** Household air pollution, unclean fuels, solid biomass, acute respiratory infections, under-five children, Tanzania

## Abstract

**Background and Aims:**

Increased risk of acute respiratory infection (ARI) in children has been linked with exposure to household air pollution (HAP) from solid biomass fuels. However, information is limited on the trend use of biomass fuels and their association with ARI among children in Tanzania. The current study analysed nationally representative data from the Tanzania Demographic Health Surveys of the years 2004, 2010, and 2015–16 to explore the prevalence of the trend of cooking fuels and ARI as well as ascertain their association among under-fives.

**Methods:**

A total sample of 20,323 under-fives were included in the current analysis. A mixed-effects multilevel logistic regression was fitted to assess the association between unclean fuels (solid biomass fuels and kerosene) and ARI among under-fives.

**Results:**

The use of solid biomass fuels has remained persistent high (98.6%) while ARI among under-fives has declined from 16% in 2004 to 9% in 2016; *p* < 0.001. Furthermore, under-fives exposed to unclean fuel combustion had a significantly higher incidence of ARI (AOR = 3.47; 95% CI, 1.31–9.21).

**Conclusion:**

Efforts should be made to switch to alternative sources of clean energy such as natural gas and biogas in Tanzania and other countries with similar settings.

## Background

About 3 billion of the world’s population relies on solid biomass fuels and kerosene as their primary source of energy for cooking, lighting, and heating [1]. A large proportion of this population lives in low- and middle-income countries (LMICs) [2]. The burden of HAP in LMICs countries is on the rise as a result of rapid population growth on the background of limited accessibility and affordability of clean fuels such as liquefied petroleum gas (LPG), natural gas, and electricity [2,3]. In Tanzania, the energy balance is considerably dominated by solid biomass fuels especially wood fuel (firewood and charcoal) accounting for about 90% of the primary source of energy for cooking, heating and lighting. Moreover, the majority of the population has low purchasing power suggesting a high preference for the cheapest source of energy – solid biomass fuels [4].

Women and children are the vulnerable populations to HAP because of their frequent exposure to particulate matter (smoke and soot) from solid biomass fuel combustion during cooking [5,6].Traditionally, women are the chef cooks in the society or majority of the households. Since young children spend a significant amount of time with their mothers, they are also exposed to cooking fuels combustion [7]. Studies have revealed that particulate matter inhaled from biomass fuel combustion has a significant negative impact on the health of mothers and their unborn babies, as well as on under-five children [3,8]. According to the World Health Organization (WHO), about half of the ARI mortality in under-fives is due to particulate matter inhaled from biomass-based household air pollution [3].

In LMICs, studies have revealed considerable effects of solid biomass fuels and kerosene on the incidence of ARI among under-fives. HAP has been shown to increase the risk of childhood ARI two-fold and responsible for 45% of all pneumonia deaths in under-fives [3]. In a systematic review and meta-analysis involving 51 studies from LMICs, the overall pooled odds ratios (ORs) showed a significant association between solid biomass fuels exposure and ARI in children (OR = 3.53; 95% CI, 1.94 – 6.43) [9]. A nationwide population-based study in Bangladesh found that 90% of under-fives exposed to solid biomass fuels had a significantly higher likelihood of ARI (AOR = 1.18; 95% CI,1.08–1.33) [10]. A comparable finding was observed in another study in Pakistan whereby the incidence of ARI was relatively higher among children from households exposed to solid biomass fuels (RR 2.6, 95% CI, 1.5–4.5) and kerosene (RR 1.9, 95% CI 1.3–2.8) [11]. Furthermore, the effect of HAP on childhood ARI has been reported by studies in sub-Saharan African countries such as Ghana [12], Zimbabwe [13], Sierra Leon [14], Ethiopia [15], and Uganda [16].

In Tanzania, for more than a decade, few small scale studies and one national-wide study have examined the effects of HAP from biomass fuels on ARI in under-five children. A study conducted in Bagamoyo town council found that under-five children and cooks had a higher likelihood of suffering from ARI 5.5 (95% CI: 3.6–8.5) than their counterpart – unexposed group [17]. Similarly, a nationwide population-based study conducted in 2007 found that under-five children exposed to HAP had twice higher odds of suffering from ARI [18]. The 2007 study was based on the analysis of a single dataset of the 2004 Tanzania Demographic Health Survey (TDHS). Moreover, the type of cooking fuels was categorized into biomass fuels and a combination of charcoal/kerosene. For the past decade, there has been limited information on the trends of the effects of cooking fuel combustion and the incidence of ARI in under-fives in Tanzania. In the current study, we used data from three subsequent nationwide population-based surveys: 2004, 2010, and 2015–16 to explore the prevalence of the trend of cooking fuels and ARI as well as ascertain their association among under-fives in Tanzania. Solid biomass fuels and kerosene are regarded as unclean fuels because they are highly polluting fuels [19]. Therefore, the current study categorized cooking fuels into clean fuels and unclean fuels.

## Methods

### Data source

The TDHSs datasets of the years 2004, 2010, and 2015–16 was used in the current analysis. The TDHSs were undertaken by Tanzania’s National Bureau of Statistics (NBS) in collaboration with the Office of the Chief Government Statistician (OCGS) Zanzibar; the Ministry of Health Community Development, Gender, Elderly and Children (MoHCDGEC) Tanzania Mainland; and the Ministry of Health (MOH) Zanzibar. The technical support for the survey was provided by ICF International under the DHS program. The TDHSs have been conducted after every four years to improve the health of the Tanzanian population. The survey collected information from a nationally representative sample of women aged 15 – 49 years in the selected households.

### Study sample and sampling technique

**Figure 1:**
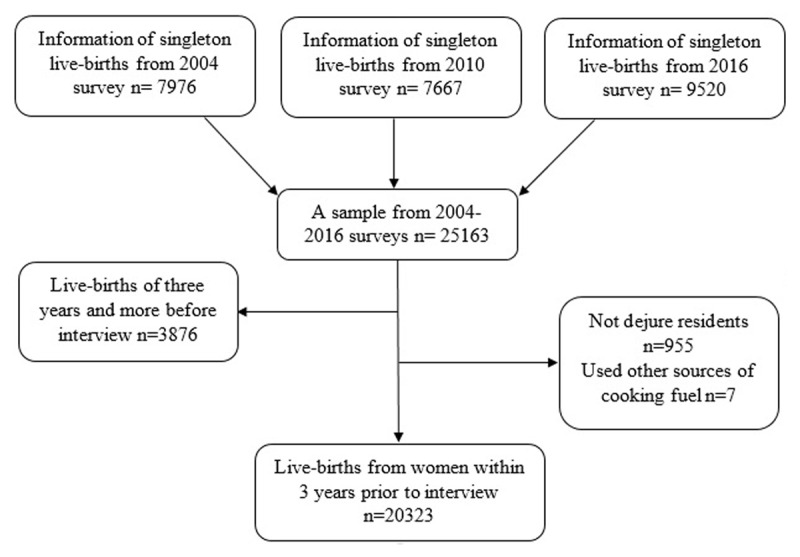
Selection of study participants.

The samples for the TDHSs were based on two-stage cluster sampling techniques. In the first stage, the primary sampling units (PSUs): a total of 475 clusters in 2004 and 2010 TDHSs and 608 clusters in 2015–16 TDHSs were selected from a sampling frame consisting of enumeration areas delineated by the 2002 and 2012 Tanzania Population and Housing Census, respectively [20]. A complete households listing was carried out in all selected clusters (PSUs) to create a sampling frame for the second stage selection of households. In the second stage, a total of 22 households were systematically selected from each cluster. Women and men aged 15–49 years who were either usual residents or visitors in the household on the night before the survey were included. The current study used information from selected eligible women who completed the women questionnaire on maternal and child health behaviour as well as their outcomes. In total information of 33,734 women who had at least one live birth in the five years preceding the survey was collected: 10329 women in 2004, 10,139 women in 2010, and 13,266 women in 2015–16 yielding an average response rate of 97%. Information regarding child health was obtained from 25,163 singleton live births babies, of which 7,976, 7,667, and 9,520 live births were from 2004, 2010, and 2015–16 TDHSs, respectively. Information from women who had at least one live birth three years and more (n = 3878) preceding the survey, not dejure residents (n = 655), and used other sources of cooking fuel (n = 7) was excluded. Finally, a total of 20,323 singleton live births information from women who had at least one live birth within three years preceding the interview dates and nested within 608 clusters were pooled from 2004, 2010 and 2015–16 surveys and analyzed (Figure 1).

## Measurement of Variables

### Outcome variable

The main outcome variable was the presence of symptoms of acute respiratory infection in the two weeks before the interview dates [21,22]. For each child, the mother was asked whether her child had the following symptoms of ARI: cough accompanied by (1) short, rapid breathing that is chest-related, and/or (2) difficulty breathing that is chest-related, plus (3) fever in the two weeks before the interview dates. The ARI symptoms were computed as a dichotomous “yes” and “no” variable. The “yes” category for a child who reported experiencing the aforementioned symptoms and the “no” category for otherwise.

### Primary explanatory variable

The primary explanatory variable was the type of cooking fuel used for indoor cooking. During the interview, mothers were asked to mention the type of fuel mainly used for cooking. The questionnaire had the following categories of fuels: electricity, liquefied petroleum gas (LPG), natural gas, biogas, kerosene, coal, lignite, charcoal, wood, straw/shrubs/grass, agricultural crop, and animal dung. In the current analysis cooking fuels were further categorized into clean fuels (electricity, LPG, natural gas, and biogas) and unclean fuels which include solid biomass fuels (charcoal, coal, lignite, wood, straw/shrubs/grass, agricultural crop, and animal dung) and kerosene [19]. Unclean fuels are regarded as highly polluting fuels [19]. This method of categorizing cooking fuels has been used in previous studies [23,24].

### Other covariate variables

Cluster variables: this includes geographical cluster zones and residence clusters. The geographical cluster zones are summarized in Table 1 below:

**Table 1:** Geographical cluster zones.


CLUSTER ZONE		REGIONS

1	Central	Dodoma and Singida

2	Coastal	Dar es salaam, Pwani, Tanga and Morogoro

3	Lake	Kagera, Mwanza, Mara, Simiyu and Geita

4	Northern Highlands	Arusha, Kilimanjaro and Manyara

5	Southern	Lindi, Mtwara and Ruvuma

6	Southern Highlands	Iringa, Mbeya, Rukwa and Njombe

7	Western	Tabora, Shinyanga and Kigoma

8	Zanzibar	Unguja North, Unguja South, Town West, Pemba North and Pemba South


The residence was categorized as “Urban” for clusters located in cities, municipalities and town councils while “Rural” for clusters that were located outside the urban areas as it was gazetted under the Local Government Act, 1982 [25].

Individual/household variables: the sex of the child was categorized as “female” or “male”. The age of the children was categorized as “neonatal” for children less than 29 days; “post-neonatal” for children aged 29 days to 11 months; and “childhood” for children aged 12 – 59 months. The age of the mother was categorized into “15–19,” “20–34,” and “35–49.” Mother’s education level was categorized into “no education,” “primary level,” and “secondary level or higher.” Mother working status was categorized as “not working,” “self-employed,” and “employed.” Father’s education level “no education,” “primary level,” and “secondary level or higher.” The number of living children was grouped as “1–2” for children whose mother had 1 or 2 living children, “3–4” for children whose mother had 3 or 4 living children, “5+” for children whose mother had 5 or more living children. The wealth index was computed based on household assets and housing characteristic information that was collected in 2004, 2010, and 2015–16 TDHSs Household Questionnaire. This questionnaire covers information about household ownership of some consumer items, ranging from a television to a bicycle or car, as well as information on dwelling characteristics such as the source of drinking water, type of sanitation facilities, and type of materials used in the dwelling construction. Each asset was assigned a weight (factor score) generated through principal component analysis, and the resulting asset scores were standardized to a standard normal distribution with a mean of 0 and a standard deviation of 1. Each household was then assigned a score for each asset, and the scores were totalled for each household. Individuals were ranked according to the total score of the household in which they resided. The distribution is then allocated into five equal categories (quintile): “poorest”, “poorer”, “middle”, “richer”, and “richest”. In the current study, the wealth index was re-categorized as “Poor” for those in the poorest and poorer quintiles; “Middle” for those in the middle quintile; and “Rich” for those in the richer and richest quintiles.

### Statistical analysis

Initially, the data from 2004, 2010, and 2015–16 TDHSs were combined to assess the change in patterns of outcome variable “ARI symptoms” and primary explanatory variable “type of cooking fuel” within the household. Then, a mixed-effects multilevel logistic regression analysis was applied to account for the hierarchical structure of the data whereby individuals or households (level 1) were nested within clusters (level 2). The multilevel models with two levels were fitted to assess the association between ARI symptoms and the type of cooking fuel adjusted by other individual or household and cluster-level variables (fixed effects). Furthermore, the cluster level random effects were estimated. A total of four models with the outcome variable “Acute Respiratory Infection” were estimated. In the Model, I (empty model) no predictor variable was added. This model showed the total variance of ARI symptoms between clusters. In Model II, only the major predictor “type of cooking fuel” was included. Model III included all individual or household variables (level 1). Model IV included both individual or household variables and cluster variables. For Models II, III, and IV the results of the fixed effects were presented as odds ratios (OR) with their corresponding confidence interval (CI), while the results of the random effects were presented as the variance. The variance random component of the models was estimated by calculating the variance of the cluster-level variations and their corresponding standard errors. The Intra-class correlation (ICC) was calculated to evaluate whether the variations in the ARI symptoms are primarily within or between the clusters [26,27]. Since the individual or household (level 1) were nested within the clusters (level 2), the Chi-square likelihood–ratio test was used to assess the difference between models. The p-values were estimated using Wald statistics and *p* < 0.05 was taken to indicate statistical significance. The statistical analyses were performed using Stata 14 (Stata Corp, College Station, TX). The “svy” set command was used to adjust for the complex sampling design used by TDHSs. All estimates were weighted to correct for non-responses and disproportionate sampling. The generalized variance inflation factor (VIF) was performed to test for multi-collinearity, which usually should not exceed 5. In this analysis no variable presented with VIF > 2.0, suggesting no suspicions for multi-collinearity.

### Ethical considerations

The TDHSs of the years 2004, 2010 and 2015–16 was approved by the Tanzania National Institute for Medical Research (NIMR), the Zanzibar Medical Ethics and Research Committee (ZAMREC) and the Institutional Review Board of ICF International in the USA. The informed consent was requested and obtained from the respondents after adequately explained about all relevant aspects of the study, including its aim and interview procedures. All respondents, who accepted to participate in the surveys were provided with a signed written informed consent.

In this accord, the present study was based on an analysis of the existing public domain of TDHSs datasets of the year 2004, 2010 and 2015–16 which are freely available online and with all participant’s names or identifier information detached. Therefore, the ethical approval for the current analysis was automatically deemed unnecessary. Moreover, permission to use the aforementioned datasets in the current study was obtained from DHS Program accessed through https://dhsprogram.com/data/new-user-registration.cfm.

## Results

### Baseline characteristics of respondents

The baseline characteristics did not differ substantially between the years 2004, 2010 and 2015–16 surveys, except for parents’ (mothers and fathers) level of education and mother’s working status. About three-quarters (74.81%) of information was from the childhood age group (11–59 months) and the majority (79.69%) of the respondents were from rural settings. The proportion of solid biomass fuels used was highly reported in all surveys: 99.58% in 2004; 98.16% in 2010; and 98.99% in 2015–16 (Table 2).

**Table 2:** Distribution of baseline characteristics and ARI by year of survey.


VARIABLE	2004 n (%) 6559 (100)	2010 n (%) 6260 (100)	2015–16 n (%) 7504 (100)	ALL n (%) 20323 (100)

**Main independent**				

**Cooking fuel**				

Cleaner fuel n (%)	28 (0.42)	63 (1.01)	29 (0.38)	120 (0.59)

Kerosene	0 (0.00)	52 (0.83)	47 (0.63)	99 (0.72)

Solid biomass	6531 (99.58)	6145 (98.16)	7428 (98.99)	20104 (98.61)

**Level 1 variables (Individual)**				

**Age period of the child**				

Neonatal	81 (1.22)	64 (1.03)	172 (2.29)	317 (1.56)

Post-neonatal	1586 (24.19)	1507 (24.07)	1725 (22.99)	4818 (23.48)

Childhood	4892 (74.59)	4689 (74.90)	5607 (74.72)	15188 (74.81)

**Sex of the child**				

Male	3252 (49.58)	3105 (49.60)	3808 (50.75)	10165 (50.02)

Female	3307 (50.42)	3155 (50.40)	3696 (49.25)	10158 (49.08)

**Age of mother**				

15–49	395 (6.02)	368 (5.87)	588 (7.84)	1351 (6.65)

20–34	5049 (76.98)	4522 (72.25)	5217 (69.52)	14788 (72.77)

35–49	1115 (17.00)	1370 (21.88)	1699 (22.64)	4184 (20.59)

**Mother education level**				

No education	1780 (27.13)	1675 (26.76)	1641 (21.87)	5096 (25.07)

Primary	4501 (68.63)	4231 (67.59)	4822 (64.25)	13554 (66.70)

Secondary/above	278 (4.23)	354 (5.65)	1041 (13.88)	1673 (8.23)

**Mother working status**				

Not working	588 (8.96)	662 (10.58)	1294 (17.24)	2544 (12.52)

Self-employed	5255 (80.13)	4501 (71.90)	4318 (57.55)	14074 (64.07)

Employed	716 (10.91)	1097 (17.52)	1892 (25.21)	3705 (18.23)

**Father education level**				

No education	1474 (22.48)	1444 (23.07)	2123 (28.29)	5041 (24.80)

Primary	4630 (70.60)	4377 (69.92)	4404 (58.69)	13411 (65.99)

Secondary/above	455 (6.92)	439 (7.01)	977 (13.02)	1871 (9.21)

**No. of living children**				

1–2	2492 (37.99)	2168 (34.63)	2902 (38.67)	7562 (37.21)

3–4	2184 (33.30)	2174 (34.73)	2317 (30.88)	6675 (32.84)

5+	1883 (28.71)	1918 (30.64)	2285 (30.45)	6086 (29.95)

**Wealth index class**				

Poor	2960 (45.13)	2936 (46.90)	3628 (48.35)	9524 (46.86)

Middle	1458 (22.23)	1445 (23.08)	1439 (19.18)	4342 (21.37)

Rich	2141 (32.65)	1879 (30.02)	2437 (32.47)	6457 (31.77)

**Level 2 variables (Cluster)**				

**Geographical zone**				

Central	566 (8.62)	606 (9.68)	551 (7.34)	1723 (8.48)

Coastal	863 (13.16)	902 (14.41)	1228 (16.37)	2993 (14.73)

Lake	1452 (22.14)	1368 (21.85)	2175 (28.99)	4995 (24.58)

Northern	581 (8.86)	482 (7.70)	674 (8.98)	1737 (8.55)

Southern	405 (6.17)	426 (6.81)	394 (5.25)	1225 (6.03)

Southern highlands	993 (15.14)	864 (13.80)	819 (10.91)	2676 (13.17)

Western	1531 (23.34)	1448 (23.13)	1349 (17.98)	4328 (21.29)

Zanzibar	168 (2.57)	164 (2.62)	314 (4.18)	646 (3.17)

**Cluster residence**				

Rural	5424 (82.69)	5133 (82.00)	5639 (75.15)	16196 (79.69)

Urban	1135 (17.31)	1127 (18.00)	1865 (24.85)	4127 (21.74)


*Note*: n and (%) represent the absolute number and percentage in the bracket, respectively.

### Trends of ARI among under-five children

**Figure 2:**
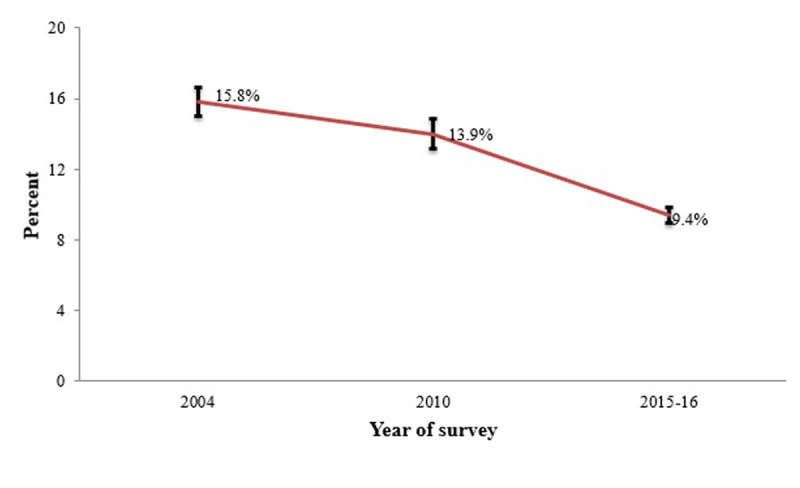
Percentage distributions of under-fives with symptoms according to the survey year, TDHS 2004–2016 (n = 20323).

Figure 2 shows the trend in the proportion of ARI among under-fives between surveys of the years 2004, 2010 and 2015–16. There was a significant decline in the prevalence of ARI from 2004 to 2010 and 2015–16 surveys: 15.8%, 13.9% and 9.4%, respectively (*P* < 0.001). However, the prevalence of ARI was relatively higher for under-fives from rich households (Supplementary Table 1).

### Association of Acute Respiratory Infections and Cooking Fuels

Table 3 presents the results of a mixed-effects multilevel logistic regression of the association between cooking fuels and ARI among under-fives. The random effects of Model I (empty model) showed significant variability in the odds of ARI among under-fives between clusters [VAR = 0.350; SE = 0.040]. Intra-Class Correlation (ICC) showed that 9.6% of the total variance in ARI among under-fives was attributed to the differences between clusters. The fixed effects of Models II, III, and IV showed that under-fives who were exposed to unclean fuels had a significantly higher likelihood of ARI than their counterparts. The final model-IV showed that under-fives exposed to unclean fuels were 3.47 times more likely to suffer from ARI than under-fives who were exposed to clean fuels. Moreover, under-fives in the post-neonatal and childhood age groups had higher odds of suffering from ARI; [AOR 16.30, 95% CI; 4.99–53.27] and [AOR 13.82, 95% CI; 4.27–44.76], respectively than those from neonatal age group. The model depicted a significant decline in the odds of ARI for the year 2016 than in the years 2010 and 2004.

**Table 3:** Multilevel logistic regression models for the association of HAP and ARI among under-fives, TDHS 2004/2010/2015–16.


VARIABLE	MODEL 1 AOR [95% CI]	MODEL 2 AOR [95% CI]	MODEL 3 AOR [95% CI]	MODEL 4 AOR [95% CI]

**Main independent**				

**Cooking fuel**				

Cleaner fuel		1.00	1.00	1.00

Unclean fuel	–	2.77 [1.21–6.35]	3.15 [1.17–8.51]	3.47 [1.31–9.21]

**Level 1 variables (Individual)**				

**Year of survey**				

2004			1.00	1.00

2010	–	–	0.85 [0.72–1.01]	0.85 [0.72–1.00]

2015–16			0.49 [0.41–0.59]	0.49 [0.41–0.58]

**Age period of the child**				

Neonatal			1.00	1.00

Post-neonatal	–	–	16.40 [5.02–53.57]	16.30 [4.99–53.27]

Childhood			13.89 [4.29–45.00]	13.82 [4.27–44.76]

**Sex of the child**				

Male			1.00	1.00

Female	–	–	0.95 [0.86–1.05]	0.95 [0.86–1.04]

**Age of mother**				

15–49			1.00	1.00

20–34	–	–	0.70 [0.56–0.86]	0.70 [0.57–0.87]

35–49			0.72 [0.54–0.96]	0.73 [0.55–0.97]

**Mother education level**				

No education			1.00	1.00

Primary	–	–	0.95 [0.81–1.11]	0.95 [0.81–1.12]

Secondary/above			1.07 [0.82–1.38]	1.05 [0.81–1.36]

**Mother working status**				

Not working			1.00	1.00

Self-employed	–	–	1.03 [0.86–1.23]	1.01 [0.83–1.22]

Employed			1.09 [0.90–1.32]	1.06 [0.87–1.29]

**Father education level**				

No education			1.00	1.00

Primary	–	–	1.20 [1.03–1.40]	1.20 [1.04–1.40]

Secondary/above			1.19 [0.95–1.47]	1.16 [0.93–1.45]

**No. of living children**				

1–2			1.00	1.00

3–4	–	–	0.92 [0.79–1.07]	0.92 [0.79–1.08]

5+			0.80 [0.67–0.97]	0.81 [0.67–0.97]

**Wealth index class**				

Poor			1.00	1.00

Middle	–	–	0.95 [0.81–1.11]	0.96 [0.83–1.12]

Rich			1.14 [0.95–1.35]	1.11 [0.92–1.33]

**Level 2 variables (Cluster)**				

**Geographical zone**				

Central				1.00

Coastal				0.85 [0.63–1.14]

Lake				0.87 [0.66–1.15]

Northern	–	–	–	0.58 [0.42–0.81]

Southern				0.90 [0.66–1.23]

Southern highlands				0.59 [0.43–0.81]

Western				0.90 [0.66–1.22]

Zanzibar				0.83 [0.62–1.11]

**Cluster residence**				

Rural				1.00

Urban		–	–	1.17 [0.96–1.43]

**The variance of random part**				

**Cluster level variance (SE)**	0.350 (0.040)	0.349 (0.040)	0.360 (0.042)	0.350 (0.045)

**Intraclass correlation (SE)**	0.0961 (0.0099)	0.0959 (0.0099)	0.0987 (0.0105)	0.0962 (0.0111)

**Akaike’s information criterion**	15258.490	15254.120	14978.590	14944.590

**Log pseudolikelihood**	–7627.245	–7623.061	–7468.295	–7443.296


## Discussion

The current study sought to explore the trend in the use of cooking fuels and the incidence of acute respiratory infection as well as ascertain their association among under-five children.

A multilevel logistic regression showed that there was a significant likelihood of suffering from ARI among under-fives exposed to unclean fuels (solid biomass and kerosene) than those exposed to clean fuels. This finding suggests that unclean fuels have an independent significant correlate with ARI even after controlling for other confounders in the model. Previous studies in LMICs countries revealed that under-five children exposed to unclean fuels (biomass fuels and kerosene) had a significantly higher likelihood of suffering from ARI than their counterparts [3,9,10,11,13,14,15,28]. These comparable findings across LMICs indicated that the impact of unclean fuels on the incidence of ARI in under-fives is of significant concern. Since the majority of the population in LMICs relies on unclean fuels as the cheapest source of energy [2], therefore, are frequently exposed to particulate matter which increases the risk of ARI [29].

In the current study, the use of solid biomass fuel has remained relatively high for the three consecutive surveys: 2004, 2010, and 2015–2016. On the other hand, the use of kerosene and clean fuels has relatively declined. People with low purchasing power prefer solid biomass fuels over kerosene and clean fuels because unclean fuels are the cheapest source of energy [30]. The use of clean fuels is highly dependent on household purchasing power [4]. In the current study, the household wealth index could indirectly reflect a corresponding purchasing power. Although the proportion of household wealth index in the three consecutive surveys has remained relatively stable a large proportion was in the poor wealth quintiles. Therefore, this can explain why there was a relative increase in the use of solid biomass fuels. Furthermore, population growth and ageing between the surveys might have add-on the number of people who prefer solid biomass fuel. In the present study, the proportion of study participants has also increased between the 2010 and 2016 surveys.

The trend in the prevalence of ARI among under-fives has significantly declined in the three surveys while the percentage use of solid biomass fuels has relatively increased. In the year 2013, the Tanzania MoHCDGEC launched a new vaccine – pneumococcal conjugate vaccine (PCV) as one of the prevention and control measures against pneumonia caused mostly by *Streptococcus pneumoniae* [31]. The steep decline in the prevalence of ARI between 2010 and 2016 surveys can be due to the introduction of PCV. Since this vaccine is specific to causative organisms, other risk factors for ARI remain in place. Improved maternal education and employment status in the present study can also explain the significant decline in the prevalence of ARI among under-fives. Additionally, studies elsewhere have revealed that improved maternal education [32]; household wealth status [32,33]; water, sanitation and hygiene [32,34]; childhood nutrition [34]; and access to healthcare services [32,33] have a significant impact in the decline of ARI among under-five children. Despite the significant decline in the prevalence of ARI between 2010 and 2016 surveys, the current prevalence remains high suggesting that the influence of solid biomass fuels is indisputable.

Furthermore, the current study found that under-fives in the post-neonatal and childhood age groups had a significantly higher likelihood of suffering from ARI than those in the neonatal age group. According to Tanzania, nursing mothers are restricted to rest during postpartum recovery for at least one month. During this period the mother-in-law or appointed family member takes the role of cooking and other housework [35]. Therefore, neonates are less directly exposed to particulate matter from biomass fuel combustion. On the other hand, during childhood age, the majority of children are less attached to their mothers as they are involved in playful activities. Therefore, they are more likely to be exposed to other predisposing factors for ARI.

The odds of ARI among under-fives were different between the three surveys. Under-fives in the 2015–16 survey had a significantly less likelihood of suffering from ARI than those in the 2010 and 2004 surveys. Since PCV was introduced in 2013, the majority of under-fives in the 2015–16 survey had likely received the vaccine which protected them against ARI caused by *Streptococcus pneumoniae*.

The current study also found that under-five Northern and Southern highlands zones of Tanzania had a significantly less likelihood of suffering from ARI than those from other zones. This finding can be explained by the fact that the Northern and Southern highlands zones had a higher coverage of childhood immunization compared to the other zones [22].

Meteorological weather conditions including rainfall, ambient temperature, and humidity can influence the occurrence of ARI besides other risk factors. Different studies elsewhere have reported mixed findings: poor and good correlation between weather conditions and ARI in particular of viral origin such as Influenza [24,25,26,27,28]. In the current study, all three surveys were conducted in the same year period: from December to May of the following year. It is a rainy season in most parts of Tanzania with a warm ambient temperature. For instance, in the 2015–16 survey, the average maximum and minimum ambient temperature were 20.1°C and 18.3°C in December; 28.7°C and 27.9°C in May [29]. Therefore, it is likely that all study participants in the three surveys were exposed to similar weather conditions.

The current study has provided a piece of evidence about the trend in the use of unclean fuels and its effects on the occurrence of acute respiratory infection among under-five children in Tanzania over the last decade. This study used a nationally representative large sample size pooled from three consecutive TDHSs 2004, 2010, and 2015–16. The pooled results showed a clear trend in the prevalence of cooking fuels and ARI among under-fives in Tanzania. Moreover, the current study stratified the child age into three age groups to find out which one was more affected. This can help to provide age-specific intervention hence reducing the unnecessary cost that would have happened if the intervention was to include all the age groups. The current study categorized cooking fuels into clean and unclean fuels (solid biomass and kerosene). This categorization can be of significance in the policy of fuel-switching because solid biomass fuels and kerosene are highly polluting fuels. Also, the current study used a mixed-effect multilevel logistic analysis to account for the effect of contextual factors and random effect variables on the ARI among under-fives.

Although the current study used pooled data from the three surveys it remains a cross-sectional design. Therefore, the temporal association between cooking fuels and ARI in under-fives cannot be derived. Additionally, the severity/acuteness of ARI cannot be assessed. The current study finding should be interpreted with care when compared with findings from other studies. Furthermore, the variables used in the current study were self-reported hence a recall bias is inevitable.

In conclusion, over the past decade, the use of solid biomass fuels has remained high while the prevalence of acute respiratory infection in under-five children has slightly declined in Tanzania. Moreover, the likelihood of ARI in under-fives due to household air pollution from unclean fuels (solid biomass and kerosene) was significantly high regardless of contextual and cluster variations factors. Efforts should be made by the government and other relevant stakeholders to introduce and impose the policy of fuel-switching: switch to alternative sources of clean energy while reducing the use of unclean fuels. Also, the government should incentivize and/or subsidize the natural gas which is available in Tanzania. Additionally, the government should continue developing and supporting collaborative projects for biogas in rural settings.

### Ethics and consent move after data accessibility statement

The TDHSs of the years 2004, 2010 and 2015–16 were approved by Tanzania’s National Institute for Medical Research (NIMR), the Zanzibar Medical Ethics and Research Committee (ZAMREC) and the Institutional Review Board of ICF International in the USA. The informed consent was requested and obtained from the respondents after adequately explained about all relevant aspects of the study, including its aim and interview procedures. All respondents, who accepted to participate in the surveys were provided with a signed written informed consent.

In this accord, the present study was based on an analysis of the existing public domain of TDHSs datasets of the year 2004, 2010 and 2015–16 which are freely available online and with all participant’s names or identifier information detached. Therefore, the ethical approval for the current analysis was automatically deemed unnecessary. Moreover, permission to use the aforementioned datasets in the current study was obtained from DHS Program accessed through https://dhsprogram.com/data/new-user-registration.cfm

## Data accessibility Statement

The datasets used in this study are available on the Demographic and Health Survey Program repository: http://dhsprogram.com/data/available-datasets.cfm.
